# Drought stress leads to systemic induced susceptibility to a nectrotrophic fungus associated with mountain pine beetle in *Pinus banksiana* seedlings

**DOI:** 10.1371/journal.pone.0189203

**Published:** 2017-12-07

**Authors:** Jennifer G. Klutsch, Simon Francis Shamoun, Nadir Erbilgin

**Affiliations:** 1 Department of Renewable Resources, University of Alberta, Edmonton, AB, Canada; 2 Natural Resources Canada, Canadian Forest Service, Pacific Forestry Centre, Victoria, BC, Canada; USDA-ARS Fort Keogh Livestock and Range Research Laboratory, UNITED STATES

## Abstract

Conifers have complex defense responses to initial attacks by insects and pathogens that can have cascading effects on success of subsequent colonizers. However, drought can affect a plant’s ability to respond to biotic agents by potentially altering the resources needed for the energetically costly production of induced defense chemicals. We investigated the impact of reduced water on induced chemical defenses of jack pine (*Pinus banksiana*) seedlings from initial attack by biotic agents and resistance to subsequent challenge inoculation with a pathogenic fungal associate of mountain pine beetle (*Dendroctonus ponderosae*), *Grosmannia clavigera*. Applications of phytohormones (methyl salicylate and methyl jasmonate) and *G*. *clavigera* were used for initial induction of defenses. Monoterpene concentrations varied with initial induction from fungal and phytohormone application while watering treatment had no effect. Seedlings treated with *G*. *clavigera* and methyl jasmonate had the greatest monoterpene concentrations compared to the control and methyl salicylate-treated seedlings. However, the monoterpene response to the challenge inoculation varied with watering treatments, not with prior induction treatments, with lower monoterpene concentrations in fungal lesions on seedlings in the low to moderate watering treatments compared to normal watering treatment. Furthermore, prior induction from phytohormones resulted in systemic cross-induction of resistance to *G*. *clavigera* under normal watering treatment but susceptibility under low watering treatment. Seedlings stressed by low water conditions, which also had lower stomatal conductance than seedlings in the normal watering treatment, likely allocated resources to initial defense response but were left unable to acquire further resources for subsequent responses. Our results demonstrate that drought can affect interactions among tree-infesting organisms through systemic cross-induction of susceptibility.

## Introduction

Conifer trees have physical and chemical defenses that act to protect them from attacks by both insects and pathogens. On top of pre-formed constitutive defenses, chemical defenses can be induced after an initial attack, which can negatively impact the performance of the attacking organism and can deter further attacks in other parts of the tree [1–2]. The initial attack by an organism can therefore have cascading effects on the success of subsequent colonizers of the same or different species [3–7]. However, drought can predispose a tree to insect attack. This can be due to reduced carbon uptake from stomatal closure and reduced photosynthetic rate (e.g., [8]), which can negatively impact the defense metabolite production [9–11] or tolerance mechanisms, such as compensatory growth [12–13]. The limitations of producing additional defense chemicals due to drought stress may therefore alter the susceptibility of the tree to not just initial attack by organisms but further attacks by different organisms. Studies demonstrating such cascading effects of water limitation on plant-mediated interactions with a community of organisms are lacking. In this study, we investigated the impact of drought on induced defenses from initial attack and resistance to subsequent biotic attacks.

Increasing incidence of drought in western and boreal forests in North America have affected the susceptibility of trees to insects and pathogens and potentially host responses to subsequent attacks [14–19]. For example, intense droughts have led to increased susceptibility of trees to attack by bark beetles, including mountain pine beetle (*Dendroctonus ponderosae*, MPB) (Coleoptera: Curculionidae, Scolytinae) [19–22]. During the recent outbreak, MPB killed millions of trees, mainly lodgepole pine (*Pinus contorta*), in western Canada and has recently spread into the novel host jack pine (*Pinus banksiana*) forests and threatens to expand through the Boreal forest to the eastern coast of North America [23–24]. Along with drought as a predisposing factor affecting tree susceptibility to MPB, there are also many additional insects and pathogens that may influence host susceptibility to MPB [3–4].

Prior attack of a conifer by an organism that leads to increased resistance to subsequent attacks has been described as systemic induced resistance (SIR) [25]. This resistant response is usually expressed at the early phases (pre-symptomatic) of an attack when the host defensive capabilities are not substantially impaired [4, 25–26]. However, as the initial attack progresses and the conifer becomes symptomatic, the defense machinery of the tree may break down and the tree expresses systemic induced susceptibility (SIS) to subsequent attacks [25]. Drought may alter this switch from SIR to SIS as resistance is dependent on the availability of resources, such as carbohydrates, in plants with a limited ability to acquire additional resources [27–28].

These induced defensive responses after a stressor are mediated by several phytohormones that act as signaling pathways, such as salicylic acid, jasmonic acid and their methylated forms. The type of induced defenses is dependent on the feeding mode of the attacking organism [11, 29–31]. For example, infection by biotrophic and hemi-biotrophic pathogens (organisms that acquire nutrients from live plant cells, and in the case of hemi-biotrophs that can move to using dead cells at later disease stages) or phloem-sucking insects triggers the plant defense signaling pathway resulting in the accumulation of methyl salicylate (MS) [11, 31–32]. Induction of the salicylic acid pathway has been shown to increase resistance to subsequent attack by pathogens and insects in several annual and perennial plant systems [31, 33–35]. However, evidence for MS-dependent defense chemical responses or the effects on subsequent resistance to attack is scarce in conifers [11, 36–38]. In contrast, the jasmonic acid signaling pathway is associated with host defense against necrotrophic pathogens (microorganisms that kill plant tissue and acquire nutrients from dead cells) and herbivorous insects [7, 11, 31]. Application of methyl jasmonate (MJ) on conifers results in anatomical changes, such as production of traumatic resin ducts, and induction of terpenoid and phenolic compound accumulation [38–42].

We used jack pine seedlings and the MPB-associated fungus *Grosmannia clavigera* (as a proxy for MPB) to investigate the impact of drought on plant induced responses elicited from multiple signaling pathways and the SIR or SIS of trees to subsequent attack. *Grosmannia clavigera* is one of several symbiotic fungi associated with MPB that eventually contribute to tree mortality and are necessary for successful beetle reproduction [43]. These pathogenic fungi block transport of water and nutrients in the tree, help the beetle overwhelm tree defenses, detoxify some defensive compounds, and even provide nutrition to developing beetles [43–46]. Importantly, *G*. *clavigera* has been shown to induce chemical changes in multiple pine species, such as increasing the concentration of monoterpenes that can be toxic to both MPB and fungi within resin filled lesions [10–12, 42, 47]. Our objectives were to: (1) determine whether the local and systemic effect of different initial induction elicitors (*G*. *clavigera*, MS, and MJ) on monoterpene defenses of jack pine depends on drought stress, and (2) examine whether the combination of prior induction of defenses from initial induction elicitors and drought stress affect jack pine responses to subsequent challenge from *G*. *clavigera*.

## Materials and methods

One-year-old jack pine seedlings (total = 200) were obtained from Pineland Forest Nursery, Manitoba, Canada, in spring of 2012 (Seed lot #0-10-04.1-I-1635). Seedlings were planted in four litre pots with Sunshine Mix #4 (Sungro, Vancouver, BC, Canada) and maintained in a greenhouse at the University of Alberta under ambient light supplemented with full spectrum lighting (light:dark of 12:12) from March 2012 to July 2013. To meet dormancy requirements, seedlings had an eight-week period of cold hardening in a cold room (4°C with light:dark of 10:14 starting 13 November 2012) before the second growth cycle. Throughout growth and dormancy periods, seedlings were watered once a day with acidified water (pH of 5.5). During the growth period and up to the time of the initiation of the watering treatments (13 May 2013), fertilizer (17 N—5 P—19 K at 175 ppm N plus periodic micronutrient fertilization) was applied weekly. During the last four-weeks, a conditioning phase was applied before cold hardening and seedlings received a different fertilization regime (8 N—20 P—30 K at 50 ppm N).

After cold hardening (mid-January 2013), the seedlings were returned to the greenhouse and growth period conditions were resumed. The seedlings were randomly divided into eight blocks with 24 seedlings per block (192 seedlings total). A three-factorial design in a randomized-block arrangement was used for the application of the following treatments: 3 levels of watering treatment (normal, moderate, and low) × 4 types of initial induction treatment (control, *G*. *clavigera* inoculation, MJ, and MS application) × 2 types of challenge inoculation with *G*. *clavigera* (non-challenged vs. challenged) (S1 Table). This design resulted in a total of eight seedlings per watering × induction × challenge treatment with each treatment combination being represented once in each block. To standardize watering treatments and prevent water from draining from pots, each pot was placed on individual pot saucers. The normal watering treatment consisted of about ~200 ml of water daily. The low watering treatment was initiated on 13 May 2013 and continued to the end of the experiment (56 days) on eight seedlings per block and consisted of the daily application of water at 10–20% volume of the normal watering treatment. The moderate watering treatment, which consisted of daily application of 30–40% volume of water of the normal watering treatment, was initiated on eight seedlings per block on 24 May 2013 to the end of the experiment (45 days). The different extent and intensity of water limitation between low and moderate watering treatments reflects the predictions from climate models for the next century that there will be an increase in drought frequency, extent, and intensity in many forest ecosystems [48–49]. Watering treatments were maintained until harvesting.

The initial induction treatments (*G*. *clavigera*, MJ, or MS) were applied on the lower third of the stem to all seedlings on 10 June 2013 (4 and 2 weeks after initiation of low and moderate watering treatments, respectively). We did not apply a wounding treatment without inoculation because experiments that used the same jack pine seedling system found smaller necrotic lesions from wounding than inoculation with *G*. *clavigera* and similar physiological, hormone, and defensive responses as non-wounded controls (e.g., [10–11, 38]). Within blocks, seedlings were arranged into nested groups by their induction treatment to minimize any possible interaction between treatments. Seedlings in the control treatment did not receive any induction treatment. The fungal inoculation involved the removal of three disks of bark (4 mm dia.) that were spaced about 2 cm apart vertically and equally distributed around the stem [38]. The wounds were immediately inoculated with an agar plug of *G*. *clavigera* (strain collected from Fox Creek, AB, 54°24’N, 116°48’W) and covered with Parafilm (Pechiney Plastic Packaging Company, USA). For the MJ and MS applications, 100 mM solutions with 0.1% (v/v) Tween 20 were applied to the bottom third of trees using foam brushes. Application of the signaling hormones was conducted in separate rooms and the solutions were allowed to dry and absorb on trees for 24 hrs before re-assembling seedlings into blocks. While there is a potential for tree volatiles induced from MJ or MS applications to interact with other trees, others have successfully used similar methods on seedling experiments (e.g., [36, 38]) and we nested induction treatments within blocks as an attempt to minimize these interactions.

On 24 June 2013, 2 weeks after initial induction, the seedlings assigned the challenge inoculation (i.e., half of the seedlings) were inoculated with *G*. *clavigera* on the middle third of the stem. The other half of seedlings were not challenged with *G*. *clavigera* inoculations and left non-challenged. The same inoculation procedure was used as the initial induction from *G*. *clavigera*. These fungal challenge inoculations were on average 17.6 cm (SE = 0.3) above the initial induction inoculations.

Two weeks after application of the fungal challenge treatment (8 July 2013), all seedlings were harvested. Bark (including phloem tissue) was separately removed from the lowest third of stems, middle third of stems, and fungal-induced necrotic and resin-filled lesions (S1 Table). The length of each lesion was also measured and averaged for each tree with shorter lesions indicating greater resistance to fungal spread [42]. All tissues were immediately placed in liquid nitrogen and stored at -40°C until processing. Tissues were separately ground using liquid nitrogen. Seedling height and stem base diameter were also measured.

### Soil water content and stomatal conductance

To monitor the effect of watering treatments, we measured soil water content and stomatal conductance every week starting the day of initial induction treatment application on two randomly selected blocks. Soil water content was measured using time-domain reflectometry with a Tektronix 1502B (Beaverton, OR, USA). The empirical equation for organic soils [50] was used to convert the apparent dielectric constant of the soil to water content. An AP4 Leaf Porometer (Delta-T Devices Ltd., Cambridge, UK) was used to measure stomatal conductance on two needles of each tree within the selected block, which was corrected for needle area and averaged per tree.

### Monoterpene analysis

Dichloromethane extracted compounds, which mainly consist of monoterpenes, were measured using established methods [4]. Briefly, ground tissue samples (100 mg) from live seedlings were extracted twice with 0.5 ml of dichloromethane and internal standard of 0.004% tridecane. Samples with solvent were vortexed for 30 s, sonicated for 10 min, and centrifuged at 16,100 rcf at 0°C for 15 min. Sample extract (1 μl) was injected into a Gas Chromatograph/Mass Spectrometer (Agilent 7890A/5062C, Agilent Tech., Santa Clara, CA, USA) equipped with a HP-INNOWax column (I.D. 0.25 mm, length 30 m) (Agilent Tech.) with He carrier gas flow at 1 ml min^-1^, and a temperature of 50°C for 0.5 min, increased to 60°C by 2°C min^-1^ and held for 1 min, increased to 120°C by 10°C min^-1^ and held for 1 min, and then increased to 250°C by 30°C min^-1^. To quantify individual and total compounds (ng mg^-1^ of fresh tissue, hereafter concentration), the following 14 standards were used: α-terpinene, γ-terpinene (Sigma-Aldrich, St. Louis, MO, USA), 3-carene, terpinolene, α-pinene, β-pinene, limonene, myrcene, camphene, *p*-cymene, 4-allylanisole (Fluka, Sigma-Aldrich, Buchs, Switzerland), bornyl acetate (SAFC Supply Solutions, St. Louis, MO, USA), and β-phellandrene (Glidco Inc., Jacksonville, FL, USA).

### Data analysis

An ANOVA with random effects for blocks and nesting of induction treatments within blocks were separately run to compare the effect of induction and watering treatments, and their interaction on monoterpene concentration and proportion within the following tissues: 1) the lowest third of the stem without fungal challenge (i.e., area of induction treatment application), 2) middle third of the stem in trees without fungal challenge (i.e., systemic to the area of induction treatment), 3) middle third of stem in trees with fungal challenge (i.e., area surrounding fungal lesions), and 4) lesions of the fungal challenge. Average lesion length from fungal challenge was also compared between induction and watering treatments (PROC MIXED in SAS, ver. 9.3). The random effect of induction treatment nesting within blocks was removed from all models because the Akaike’s information criterion was lower when accounting for blocks alone. Where the interaction term was not significant, it was removed from the model. Tree height and stem base diameter were not significant covariates in any models and thus were not included in the final analyses. Where interaction terms were significant, partial ANOVA models with blocking were used to determine the effect of induction treatment on a response variable at each watering treatment level. Tukey’s HSD tests were performed for multiple comparisons among induction treatments. A contrast statement was used to compare monoterpene concentration in control seedlings to those treated with MJ or MS. At each measurement date for soil water content and stomatal conductance, an ANOVA with blocking was used to test whether there was an effect of watering treatment. To meet assumptions of normality and homogeneity of variance, monoterpene concentration and lesion length were natural log transformed. Back-transformed least square means with 95% confidence intervals are presented. Non-metric multidimensional scaling (NMDS) with Bray-Curtis distance and perManova with blocking were used to see whether the profile of monoterpenes varied with induction and watering treatments (R software, ver. 3.2.1). Significance levels of α = 0.05 and α = 0.10 along with p-values where applicable are reported for ANOVAs, perManovas, and Tukey’s HSD tests. This is because of a relatively low sample size for some treatments that incurred seedling mortality. Therefore, the results should be interpreted as an initial indication in a greenhouse environment of treatment effects on defense responses in seedlings.

## Results

### Soil water content and stomatal conductance

**Fig 1:**
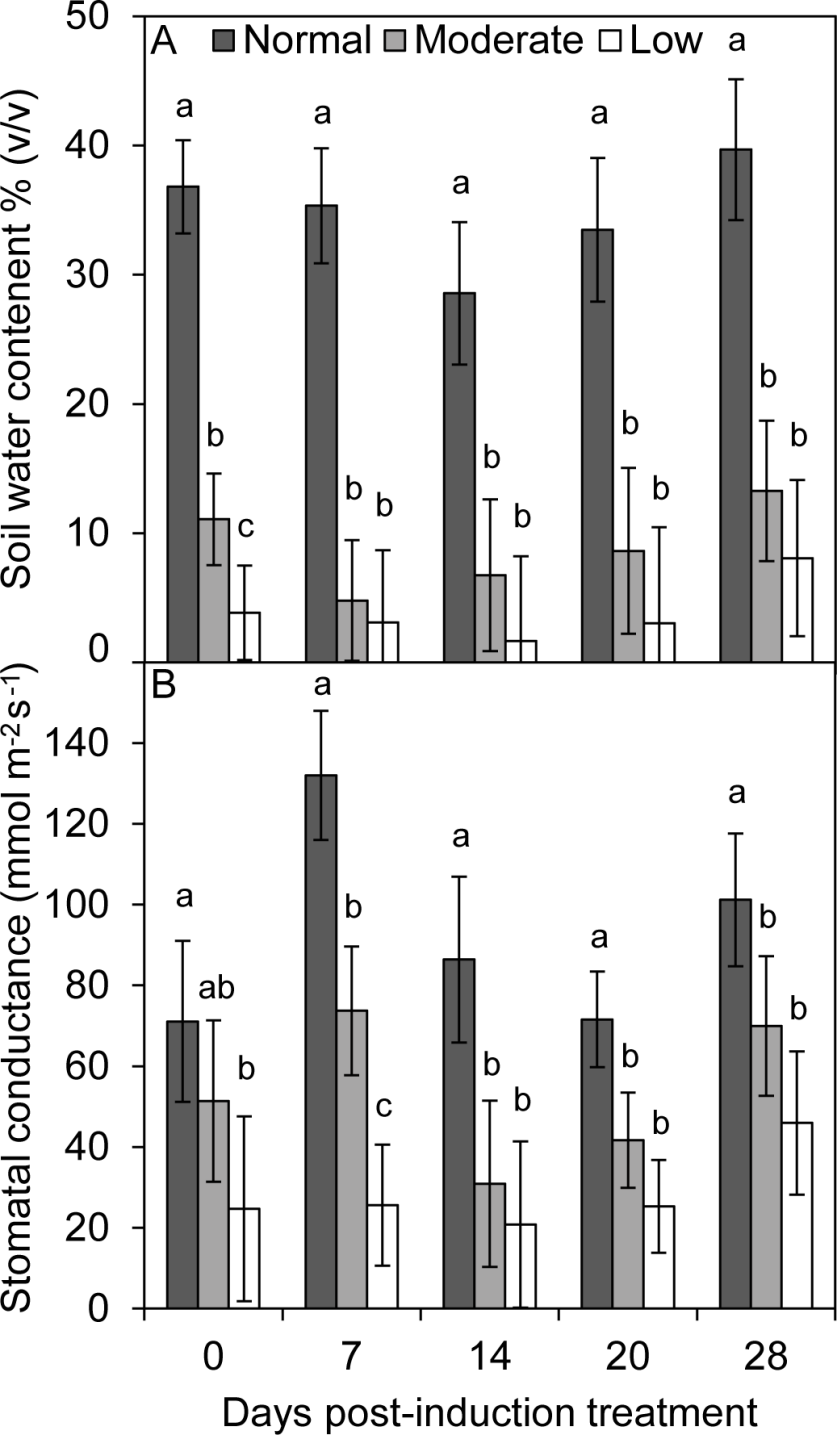
Mean (A) soil water content and (B) stomatal conductance of *Pinus banksiana* seedlings at three levels of water treatment (normal, moderate, and low) from the time of application of induction treatments (Day 0) to time of tissue sampling (Day 28). Error bars are 95% confidence intervals. Within sampling date, different letters denote significant difference among treatments using Tukey’s HSD test (α = 0.05).

At the time of induction treatment application, the soil water content was almost three and 10 times lower in the moderate and low watering treatments than in the normal watering treatment, respectively (Fig 1A). The differences between normal and low watering treatments were maintained 28 days after the application of the induction treatment until the time of tissue sampling. Furthermore, trees in the low watering treatment had almost three times lower stomatal conductance than trees with normal watering treatment at the time of induction treatment application (Fig 1B). Similarly, stomatal conductance was consistently lower in trees with low and moderate watering treatments compared to trees with normal watering treatments at the time of tissue sampling.

### Effect of water availability on local response to induction treatment

**Fig 2:**
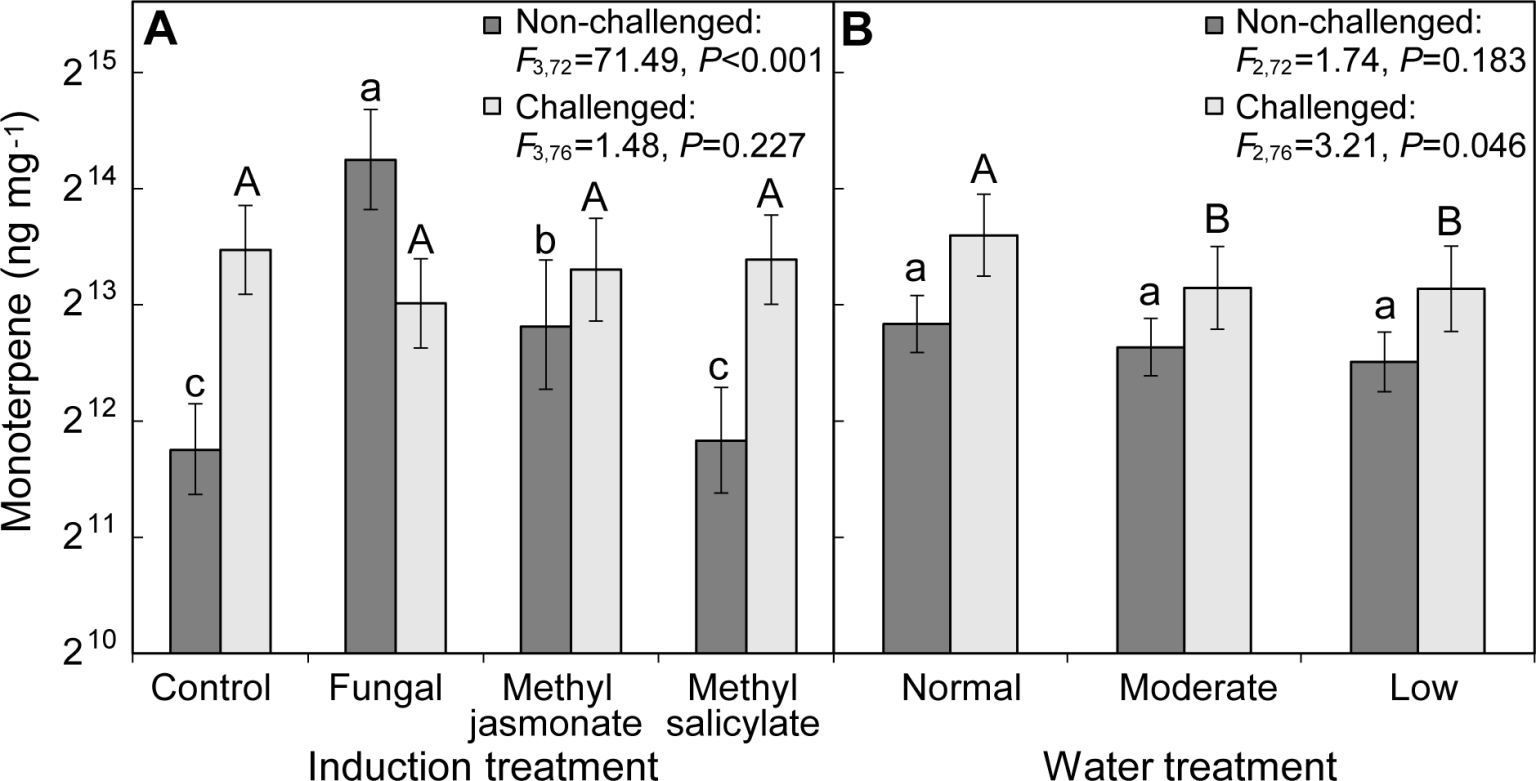
Effect of (A) initial induction (across all watering treatments) and (B) watering treatment (across all induction treatments) on monoterpene concentrations local to the site of induction treatment (dark bars) and fungal challenge (light bars) application from bark on *Pinus banksiana*. Induction treatment monoterpene concentrations are from the lower third of non-challenged seedlings at the site of initial induction treatment application for methyl jasmonate, and methyl salicylate, and within the lesions caused by inoculation by *Grosmannia clavigera*. In non-challenged seedlings (dark bars), differences among induction treatments are indicated by lowercase letters (α = 0.05), and for the fungal challenge treatment (light bars) differences among induction treatments are denoted with uppercase letters (α = 0.10). Error bars are 95% confidence intervals. The y-axis is shown in log scale.

Seedlings treated with MJ experienced high mortality, with the final number of live trees in the normal, moderate and low watering treatments being 6, 5, and 4, respectively. There was no mortality of seedlings in the other induction treatments. Across all watering treatments, total monoterpene concentration in the bark of the lower third of seedlings (at the site of induction treatment application) varied with initial induction treatment (Fig 2A). In contrast, across all induction treatments, monoterpenes did not vary by watering treatments in the seedlings (Fig 2B). There was no interaction effect between induction and watering treatments on monoterpene responses. The lesions had on average 5.6 times greater concentration of total monoterpenes than in control trees and was also greater than bark treated with signaling hormones (Fig 2A). Four monoterpenes (α-pinene, β-pinene, β-phellandrene, camphene) were also greater in the lesions than in trees with other induction treatments. Furthermore, lesions had greater concentrations of myrcene, limonene, γ-terpinene and *p*-cymene than control and MS treated trees, but not MJ treated trees. The bark treated with MJ had two times greater concentration of total monoterpenes, along with greater concentration of five monoterpenes (α-pinene, β-pinene, β-phellandrene, camphene, myrcene), compared to control trees and MS treated bark. The concentration of total monoterpenes in bark treated with MS was the same found in control trees.

Across all watering treatments on the lowest third of trees, the relative proportion of individual monoterpenes varied with induction treatment, not with water treatment. There was proportionally more β-pinene in lesions (49.3%, CI_95%_ = 47.2–51.4%) compared to control trees (38.1%, CI_95%_ = 36.1–40.2%), MJ (44.4%, CI_95%_ = 41.8–47.0%), and MS (39.1%, CI_95%_ = 37.0–41.2%) treated trees (*F*_3,72_ = 24.0, *P*<0.001). Furthermore, the proportion of camphene in lesions (1.5%, CI_95%_ = 1.3–1.6%) was greater than in control (1.2%, CI_95%_ = 1.0–1.3%) and MS (1.1%, CI_95%_ = 1.0–1.3%) treated trees (*F*_3,72_ = 3.93, *P* = 0.012). In contrast, lesions had the lowest percent proportion of both myrcene (1.2%, CI_95%_ = 0.0–2.4% [*F*_3,72_ = 2.83, *P* = 0.044]) and β-phellandrene (0.9%, CI_95%_ = 0.8–1.0% [*F*_3,72_ = 40.78, *P*<0.001]) compared to control trees (3.5%, CI_95%_ = 2.3–4.7%, and 1.3%, CI_95%_ = 1.2–1.4%, respectively). Lesions on average also had the lowest proportion of β-phellandrene compared to trees treated with signaling hormones.

Only the phenylpropanoid 4-allylanisole varied in the bark of the lower third of trees with watering treatment (*F*_2,72_ = 5.61, *P* = 0.005) along with induction treatment (*F*_3,72_ = 3.80, *P* = 0.014). The bark treated with MJ had 12 times greater concentration of 4-allylanisole (2.4 ng mg^-1^ [CI_95%_ = 1.7–5.6 ng mg^-1^]) compared to lesion tissue (0.2 ng mg^-1^ [CI_95%_ = 0.1–0.3 ng mg^-1^]), but was not different than control or MS treated bark. Also, across all induction treatments, trees in the moderate watering treatment had more than seven times greater 4-allylanisole concentration compared to trees in the low watering treatment (*t* = 3.24, *P* = 0.005), and four times more than trees in the normal watering treatment (*t* = -2.33, *P* = 0.058).

**Fig 3:**
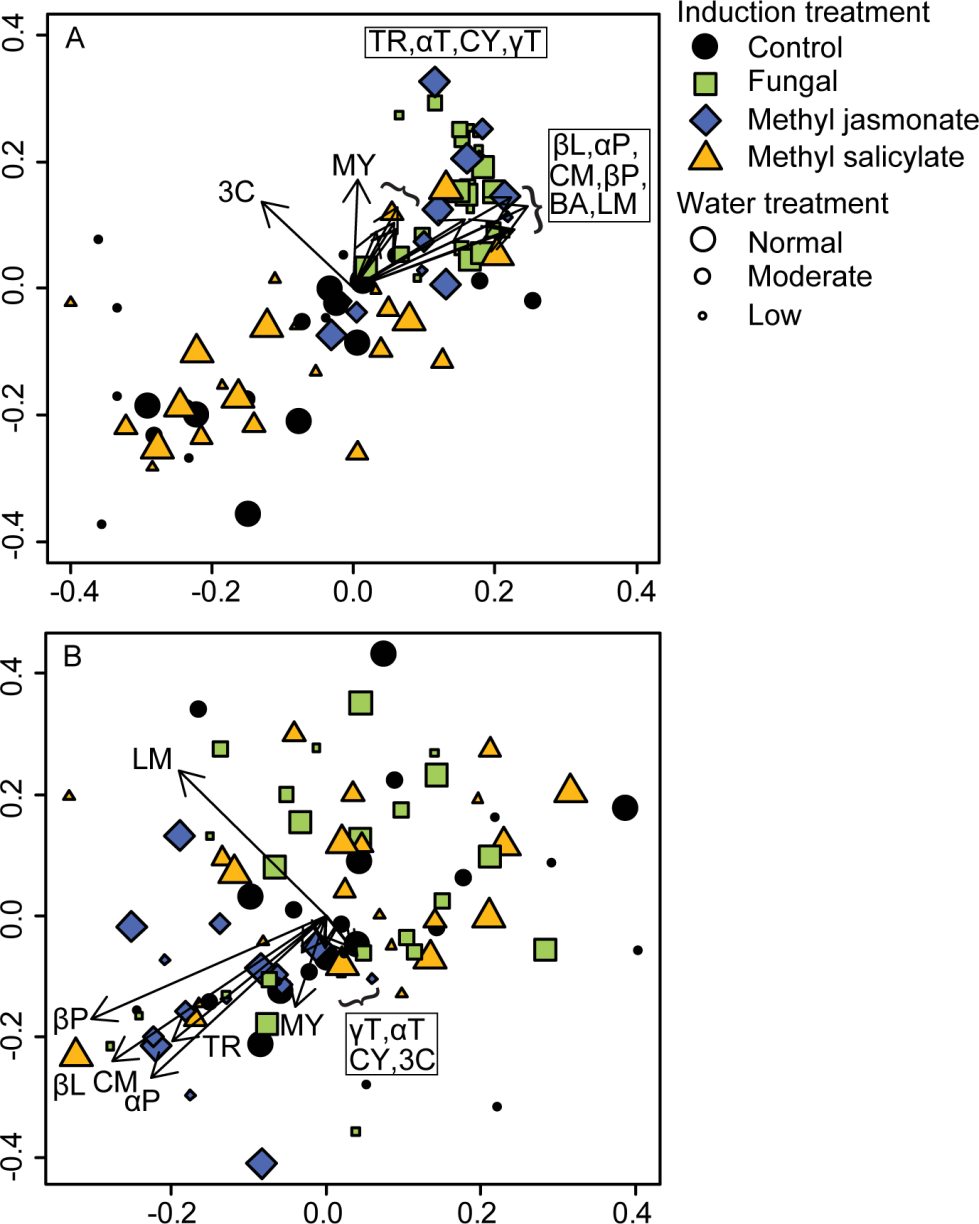
Effect of induction and watering treatments on the profile of monoterpene accumulations in bark of *Pinus banksiana* seedlings (A) at the site of the initial induction treatment application (i.e., the lower third of tree for control, methyl jasmonate and methyl salicylate treated trees and lesion tissue where *Grosmannia clavigera* was inoculated) and (B) above the induction treatment. Non-metric multidimensional scaling with Bray-Curtis distance ordination was used to analyze relationships. Significant monoterpene compounds are represented by overlaid vectors with direction indicating association with corresponding induction and watering treatments (α = 0.05; see S2 Table for correlations and *P*-values). Longer vectors show stronger correlations with the ordination configuration (i.e., axes 1 and 2). The minimum stress was: (A) 0.04 and (B) 0.17. Abbreviations for monoterpenes: αP = α-pinene, CM = camphene, βP = β-pinene, 3C = 3-carene, MY = myrcene, αT = α-terpinene, LM = limonene, βL = β-phellandrene, γT = γ-terpinene, CY = *p*-cymene, TR = terpinolene, CP = camphor, BA = bornyl acetate, and 4A = 4-allylanisole.

The monoterpene profile at the lowest third of trees varied with induction treatment (perManova *F*_3,73_ = 40.93, *P* = 0.001) and not with watering treatment (*F*_2,73_ = 1.77, *P* = 0.156). This pattern is illustrated in the NMDS analysis where the concentrations of many monoterpene compounds (represented by arrow vectors) were positively associated with trees treated with *G*. *clavigera* or MJ (Fig 3A, S2 Table).

### Effect of water availability and induction treatment on local response to fungal challenge

For seedlings challenged with *G*. *clavigera* after initial induction, mortality was high in the ones treated with MJ, with the final number of live trees in the normal, moderate and low watering treatments being 7, 6, and 4, respectively. There was no mortality of seedlings in the other induction treatments. The accumulation of total monoterpenes in lesions formed by the fungal challenge inoculation did not vary with initial induction treatment (Fig 2A) but instead only varied with watering treatment (Fig 2B). There was no interaction effect between induction and watering treatments on monoterpene responses. Tukey’s HSD tests (α = 0.1) revealed that the concentrations of total monoterpenes (Fig 2B) and camphene (*F*_2,76_ = 2.54, *P* = 0.086) tended to be greater in seedlings in the normal watering treatment compared to moderate and low watering treatments. In addition, a Tukey’s HSD test (α = 0.05) revealed that the concentration of β-pinene was 55% greater in normal watering treatment compared to trees in low watering treatment (*F*_2,76_ = 4.67, *P* = 0.012). Similarly, β-pinene made up 50.1% (CI_95%_ = 48.3–51.9%) of total monoterpenes in seedlings in the normal watering treatment, which was significantly greater than in the low watering treatment (45.0%, CI_95%_ = 43.1–46.9%; *F*_2,76_ = 7.48, *P* = 0.001). In contrast, while the concentration of 3-carene also significantly varied with watering treatment (*F*_2,76_ = 3.59, *P* = 0.033) across all induction treatments, a Tukey’s HSD test (α = 0.1) showed that trees treated with moderate and low watering treatments had greater concentrations than normal watering treatment. There was also an effect of induction treatment on one dichloromethane extracted compound, bornyl acetate. Bornyl acetate concentrations were nearly four times greater in the fungal challenge lesions of MJ treated seedlings compared to control trees, while MJ and fungal induction treated seedlings had the same concentration of bornyl acetate in the fungal challenge lesions (*F*_3,76_ = 4.63, *P* = 0.005). In a multivariate analysis of the profile of monoterpenes in the fungal challenge lesions, the concentrations varied by treatment (perManova *F*_3,88_ = 2.23, *P* = 0.039) and modestly varied by water treatment (perManova *F*_2,88_ = 2.04, *P* = 0.067).

### Effect of water availability on systemic response to induction treatment

**Fig 4:**
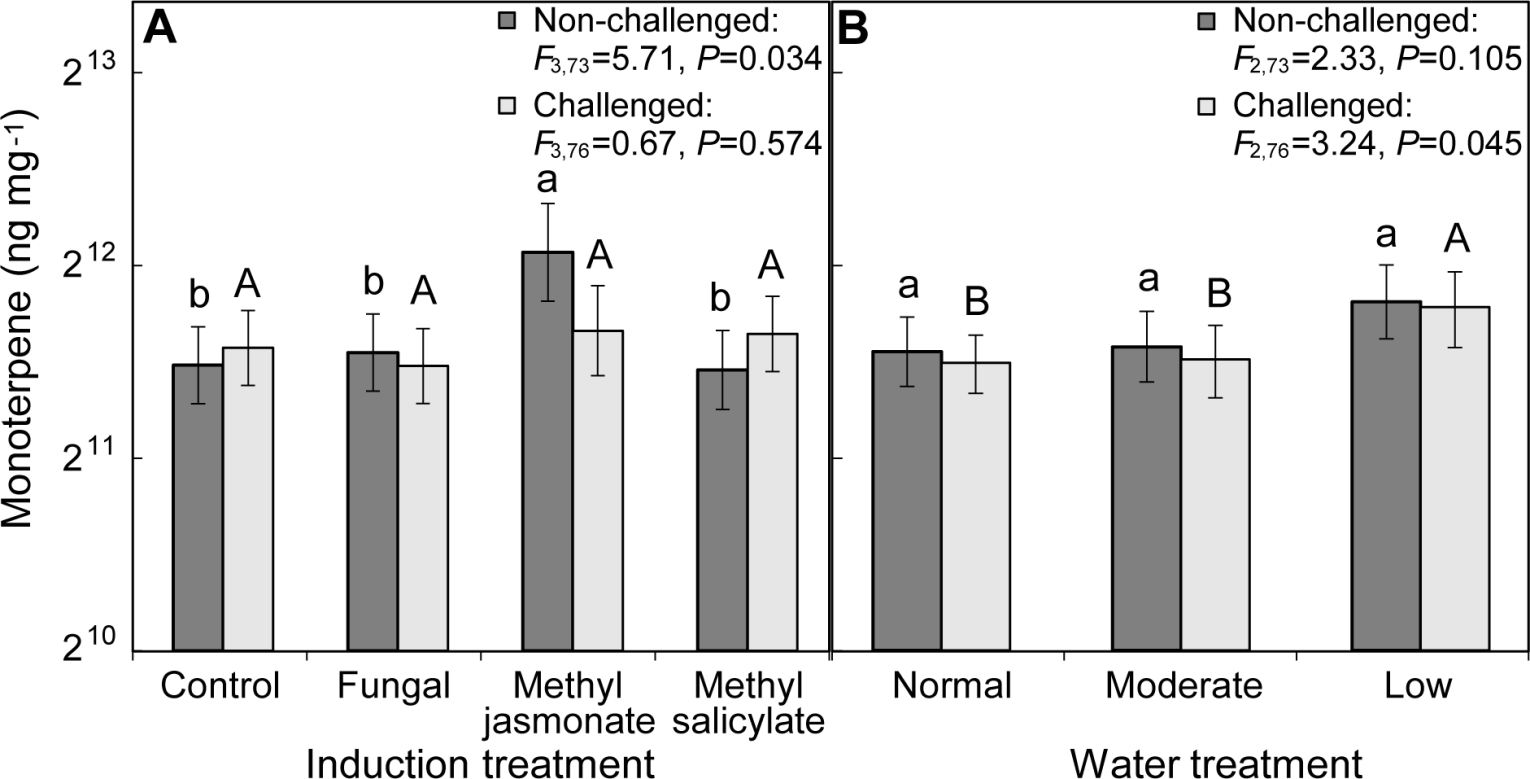
Effect of (A) initial induction and (B) watering treatment on monoterpene concentrations above the site of induction treatment (dark bars) and outside the fungal challenge lesions (light bars) from bark on *Pinus banksiana*. Induction treatment monoterpene concentrations are from the middle third of non-challenged seedlings above the site of initial induction treatment application. In non-challenged seedlings (dark bars), differences among induction treatments are indicated by lowercase letters (α = 0.05), and for fungal challenge treatments (light bars) differences among induction treatments are denoted with uppercase letters (α = 0.10). Error bars are 95% confidence intervals. The y-axis is shown in log scale.

Bark taken from above the induction treatment application area (i.e., represents the systemic response in the middle third of tree) of MJ treated trees had 1.5 times greater concentration of total monoterpenes compared to controls (Fig 4A). Five monoterpenes (α-pinene, β-pinene, β-phellandrene, camphene, and myrcene) were also highest in the MJ treated trees relative to other induction treatments and control. There was no effect of watering treatment on the systemic response to induction of monoterpene concentrations (Fig 4B).

The monoterpene profile above the induction treatment site also varied with induction treatment (perManova *F*_3,74_ = 3.27, *P* = 0.004) and not with watering treatment (*F*_2,74_ = 1.49, *P* = 0.186). Most monoterpene compounds were positively associated with MJ treated trees (Fig 3B). The relative composition of individual monoterpenes did not vary with induction or watering treatment.

### Effect of water availability and induction treatment on systemic response to fungal challenge

In the defensive area surrounding the lesions caused by the fungal challenge inoculation there was not an effect of initial induction treatment on concentration of total monoterpenes (Fig 4A) but instead an effect of watering treatment (Fig 4B). There was no interaction effect between induction and watering treatments on monoterpene responses. From Tukey’s HSD tests (α = 0.10), there was 22% more total monoterpenes (Fig 4B) and 25% more camphene in seedlings in the lowest watering treatment compared to normal watering treatment.

In contrast, the relative proportions of individual monoterpenes were not related to watering treatment. Instead, the relative proportion of two monoterpenes varied with initial induction treatment; methyl jasmonate treated trees had greater percent composition of β-pinene (42.5%, CI_95%_ = 38.5–46.4%) compared to fungal treatment (34.8%, CI_95%_ = 31.6–38.0%; *F*_3,76_ = 3.12, *P* = 0.031) and greater β-phellandrene (1.4%, CI_95%_ = 1.3–1.5%) than control seedlings (1.2%, CI_95%_ = 1.1–1.3%; *F*_3,76_ = 2.88, *P* = 0.041).

Furthermore, the profile of the concentration of all monoterpenes did not differ with induction treatments but was affected by watering treatments in the defensive zone around the fungal challenge lesions (perManova *F*_2,88_ = 2.22, *P* = 0.039). Concentrations of some compounds (i.e., β-phellandrene, camphene, β-pinene, α-pinene, 4-allylanisole) were positively correlated to the ordination configuration associated with seedlings in low watering treatment (S2 Table, S1 Fig).

### Effect of water availability and induction treatment on resistance to fungal challenge

**Fig 5:**
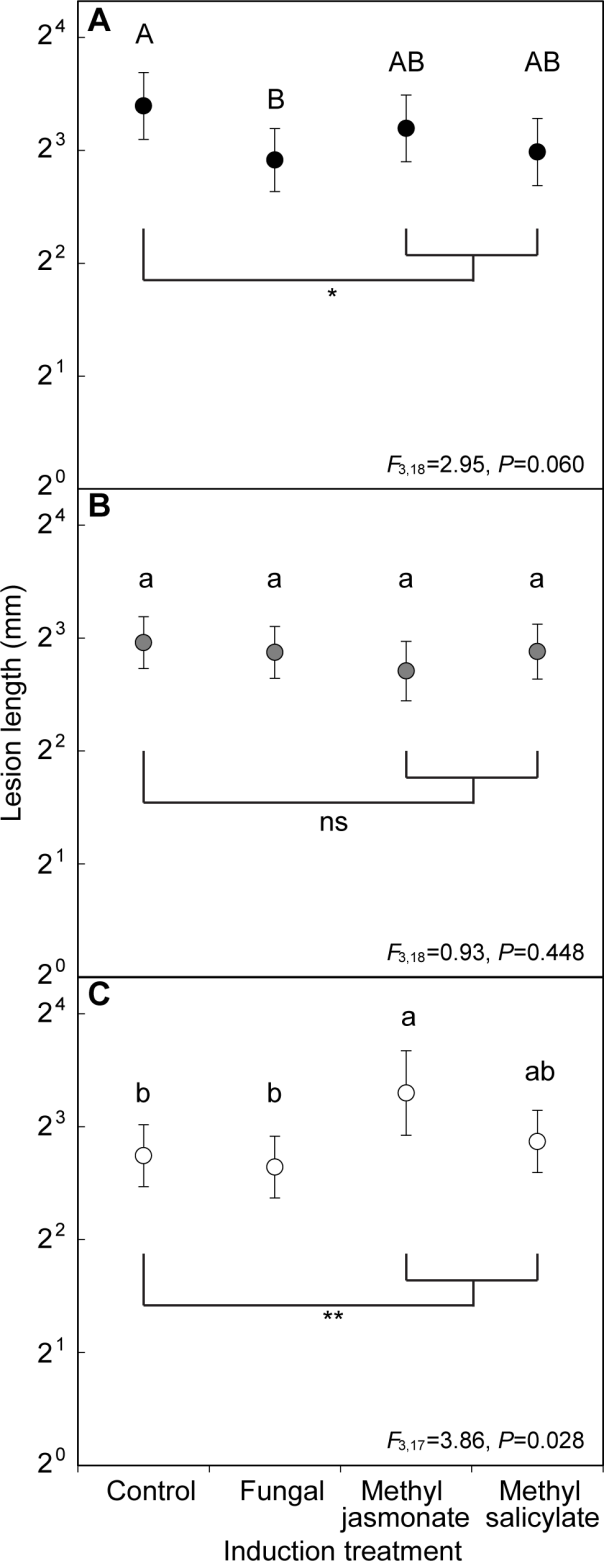
Differences in lesion lengths induced from *Grosmannia clavigera* challenge among induction treatments in *Pinus banksiana* treated with different watering treatments: (A) normal, (B) moderate, and (C) low. Different lower case letters (α = 0.05) and upper case letters (α = 0.10) denote significant difference among induction treatments using Tukey’s HSD test. Brackets with * and ** denote where control is significantly different from signaling hormone induction treatments at α = 0.10 and α = 0.05, respectively. Error bars are 95% confidence intervals. The y-axis is shown in log scale.

The effect of induction treatment on lesion lengths depended on the watering treatment (*F*_6,67_ = 2.72, *P* = 0.020; Fig 5). Control seedlings in the normal watering treatment had significantly greater lesion length compared to control seedlings in the low watering treatment (Tukey’s HSD test *P* = 0.017), while lesion length for seedlings with induction treatments did not vary with watering treatment. From partial models testing the effect of induction treatment for each watering treatment separately, we detected modestly shorter lesions from fungal challenge in seedlings initially treated with fungal inoculation in the normal watering treatment compared to control seedlings (Fig 5A). Furthermore, seedlings in the normal watering treatment and treated with the signaling hormones tended to have shorter lesions than control seedlings (*F*_1,18_ = 3.68, *P* = 0.071). Lesion lengths did not vary with induction treatment for seedlings under the moderate watering treatment (Fig 5B). In contrast, seedlings in the low watering treatment and treated with MJ had significantly longer lesions than control (Tukey’s HSD test *P*<0.046, Fig 5C). Similarly, seedlings treated with signaling hormone treatments had longer lesions than control trees (*F*_1,17_ = 5.16, *P* = 0.036).

## Discussion

Our results demonstrate that water availability affects expression of SIR in jack pine seedlings to a bark beetle-associated necrotrophic fungus. When seedlings were only subjected to one induction event, low water availability had no effect on monoterpene accumulation in jack pine-induced defenses after initial induction from fungal inoculation or phytohormone application. However, SIR and SIS for *G*. *clavigera* infection showed dependence on water availability with jack pine seedlings expressing resistance to subsequent inoculation with *G*. *clavigera* under normal watering treatment, but susceptibility under the low watering treatment. These results extend the SIR hypothesis to include environmental stress as conditions altering plant induced responses to subsequent attack.

The type of induction agent had an impact on tree response and the subsequent expression of SIR or SIS. At initial induction, the local and systemic response to MJ and MS differed, with a greater response in monoterpene concentration to MJ treatment. This is in contrast to Erbilgin and Colgan [38], which did not find a difference in monoterpene response to MJ and MS application in jack pine seedlings. However, in this study after challenge with *G*. *clavigera*, monoterpene levels of seedlings initially treated with MJ or MS were the same as control seedlings. Initial inoculation by *G*. *clavigera* resulted in lower lesion length of the second inoculation with the same fungus under normal watering treatment but there was no change under the low water. In contrast to the initial induction with *G*. *clavigera*, the initial application of the phytohormones resulted in induced resistance or susceptibility under normal or low watering treatments, respectively. These results show that tree responses shortly after an initial induction may be driven by the availability of water to seedlings. However, it is not known whether the modest cross-induction of resistance to *G*. *clavigera* inoculation under normal water conditions in this study may impact the success of MPB. Additional studies that look at systemic induced resistance to MPB in mature trees should be conducted.

In model plant systems, MS has been shown to interfere with jasmonic acid accumulation [51–52]. Therefore, the MS treatment in our study should have led to increased susceptibility to the nectrotrophic pathogen *G*. *clavigera*, which is sensitive to jasmonic acid-dependent defenses [11]. However, we found no evidence of antagonistic cross-talk between salicylic pathway and the potential jasmonic acid pathway induced from fungal inoculation, supporting earlier results in this [53] and other plant systems [54–55]. If negative cross-talk does not occur in jack pine, then the attack by a biotrophic organism (as simulated in this study by exogenous application of MS) or the accumulation of endogenous salicylic acid found in jack pine under water stress [11] may not negatively impact the defensive response against *G*. *clavigera*. Differential regulation and coordination of these and other defense pathways may also be important for fine-tuning induced resistance to multiple organisms [36, 51, 53–55].

While drought can increase susceptibility of plants to insects and pathogens [56], we found no evidence of water treatment effect on induced defense compound concentrations from initial induction treatment. Similarly, Erbilgin et al. [57] found no appreciable effect of water limitations on concentrations of induced defense compounds to *G*. *clavigera* inoculation in mature jack pine trees. These results indicate that jack pine trees may invest in defenses to initial attacks, even during time of moisture stress. López-Goldar et al. [58] similarly found that feeding on bark tissue by weevils induced the same host defense response in carbon-starved young pines as trees grown in full light. However, if biotic stress continuously occurs, only plants with available resources can keep producing defense chemicals and plants with limited resources failed to do so as the production of secondary metabolites, including monoterpenes, is metabolically costly [25, 59–60]. We found that after the initial defense responses, the seedlings with limited water had lower monoterpene responses in the challenge lesion from *G*. *clavigera* than seedlings with normal water. We suspect that the initial response is likely driven by the non-structural carbohydrates stored in plant tissues; however, continued production of defenses may require allocation of newly synthesized carbohydrates [10, 12, 61–64] if stored carbohydrates are exhausted by the initial attacks.

In contrast, at the systemic level, tree response to fungal challenge in the lower watering treatment resulted in higher monoterpene concentrations than in seedlings in the normal or moderate watering treatments, demonstrating that the effect of water availability on the cross-induction of defense responses was dependent on tissue type. Monoterpene concentrations in lesions formed from *G*. *clavigera* challenge were more than three times greater than in the defensive zone, suggesting that lesions are more energetically costly in terms of secondary metabolism than surrounding tissue [12]. This monoterpene response could also be due to the mechanical wounding from inoculation, as this research did not include wounding controls. However, the presence of treatment effects from fungal inoculation together with previous research [10, 38] that found that wounds in jack pine seedling had lower monoterpene concentrations than *G*. *clavigera* inoculated seedlings indicates that the seedlings in this study responded to *G*. *clavigera* and not just the wound. In the energetically costly fungal challenge lesions, cross-induced host responses are potentially limited by the compromised ability to acquire additional resources in drought-impacted plants.

While we found that the induced response to *G*. *clavigera* challenge in jack pine, as measured by lesion length, was positively impacted by water availability (i.e., seedlings in normal watering treatment had longer lesions than trees in the low watering treatment), this does not necessarily mean that reduced water availability made seedlings more resistant to fungal challenge. Others have documented a similar pattern of shorter lesion lengths after an initial inoculation of a bark beetle-associated fungus on drought stressed pine seedlings [11, 65–66]. This pattern may be due to slower *G*. *clavigera* growth and lesion development in low water conditions compared to normal water [11, 67]. Furthermore, drought stressed lodgepole pine × jack pine hybrids down-regulated a subset of defense-associated genes when infected by *G*. *clavigera* [66]. Therefore, in comparison with the control induction treatment within each watering treatment, the application of the initial phytohormone stress elicitors in our study resulted in a SIS response (i.e., the cross-induction of susceptibility) to *G*. *clavigera* in the low watering treatment compared to seedlings with normal water availability.

## Conclusion

In combination with the projected changes in drought patterns in western North American conifer forests, host tree responses to limited water resources may potentially contribute to their susceptibility to insect infestations including MPB. While low-level drought conditions may lead to slight resistance to bark beetle attacks in other study systems [19], this and other studies did not confirm any resistant response to *G*. *clavigera* in jack pine seedlings under reduced water availability [10–11]. We do not think this is due to an ontogenic effect as Erbilgin et al. [57] also reported similar results in mature jack pine trees to *G*. *clavigera* inoculations in low water conditions. However, because multiple organisms can also attack trees during drought conditions, low water availability can also potentially affect inter-species interactions among tree-infesting organisms through SIS. Our results demonstrate that jack pine seedling response to multiple attackers can be drought dependent. These interactions may impact jack pine susceptibility to the expansion of MPB. Furthermore, information on drought’s effect on induced resistance in conifers will be important to integrate into phenotype selection and tree breeding programs to sustain forest ecosystems.

## Supporting information

S1 Table Experimental design and sample location on tree stem. The initial induction treatments were applied on lower third of trees as follows: No application of induction agent (Control), inoculation with *Grosmannia clavigera* (Fungus), application of methyl jasmonate (MJ), and methyl salicylate (MS). Fungal challenge treatment involved the inoculation of *G*. *clavigera* on middle third of trees at two weeks after initial induction. Local tree response at the site of application of initial induction or fungal challenge treatments. Systemic tree responses are distal to the site of application of initial induction or fungal challenge treatments.
(DOCX)

S2 Table Correlations (r) and significance (*P*-value) of vector monoterpene accumulations from non-metric multidimensional scaling analyses used to visualize relationships with induction and watering treatments in the bark of *Pinus banksiana* seedlings in Fig 3 and S1 Fig. (DOCX)

S1 Fig Effect of induction and watering treatments on the profile of monoterpene accumulations in bark of *Pinus banksiana* seedlings outside the *Grosmannia clavigera* challenge lesions. Non-metric multidimensional scaling with Bray-Curtis distance ordination was used to analyze relationships. Significant monoterpene compounds are represented by overlaid vectors with direction indicating association with corresponding induction and watering treatments (α = 0.05). Longer vectors show stronger correlations with the ordination configuration (i.e., axes 1 and 2). The minimum stress was 0.14. See S2 Table for abbreviations for monoterpenes, correlations, and *P*-values.
(TIF)
