# Supplementary material for: Drought stress leads to systemic induced susceptibility to a nectrotrophic fungus associated with mountain pine beetle in Pinus banksiana seedlings
Source: PLoS One. 2017 Dec 7;12(12):e0189203. doi: 10.1371/journal.pone.0189203 (PMC5720781; doi:10.1371/journal.pone.0189203)
Supplement: S1 Table — The initial induction treatments were applied on lower third of trees as follows: No application of induction agent (Control), inoculation with Grosmannia clavigera (Fungus), application of methyl jasmonate (MJ), and methyl salicylate (MS). Fungal challenge treatment involved the inoculation of G. clavigera on middle third of trees at two weeks after initial induction. Local tree response at the site of application of initial induction or fungal challenge treatments. Systemic tree responses are distal to the site of application of initial induction or fungal challenge treatments. (DOCX) [file pone.0189203.s001.docx]

|  |  | Water Treatment | | | | | |
| --- | --- | --- | --- | --- | --- | --- | --- |
|  |  | Normal | | Moderate | | Low | |
| Initial Induction | Fungal Challenge | Local | Systemic | Local | Systemic | Local | Systemic |
| Control | Non-challenged | Phloem and lesion from lower third of tree | Phloem from middle third of tree | Phloem and lesion from lower third of tree | Phloem from middle third of tree | Phloem and lesion from lower third of tree | Phloem from middle third of tree |
| Fungus | Non-challenged |  |  |  |  |  |  |
| MJ | Non-challenged |  |  |  |  |  |  |
| MS | Non-challenged |  |  |  |  |  |  |
| Control | Challenged | Lesion from middle third of tree |  | Lesion from middle third of tree |  | Lesion from middle third of tree |  |
| Fungus | Challenged |  |  |  |  |  |  |
| MJ | Challenged |  |  |  |  |  |  |
| MS | Challenged |  |  |  |  |  |  |
