# Supplementary material for: Drought stress leads to systemic induced susceptibility to a nectrotrophic fungus associated with mountain pine beetle in Pinus banksiana seedlings
Source: PLoS One. 2017 Dec 7;12(12):e0189203. doi: 10.1371/journal.pone.0189203 (PMC5720781; doi:10.1371/journal.pone.0189203)
Supplement: S2 Table — (DOCX) [file pone.0189203.s002.docx]

|  | Non-challenged seedlings | | | | Challenged seedling | |
| --- | --- | --- | --- | --- | --- | --- |
|  | Lower third of seedling (Fig. 3A) | | Middle third of seedling (Fig. 3B) | | Outside the fungal challenge lesions (S2 Figure) | |
|  | r | *P*-value | r | *P*-value | r | *P*-value |
| α-pinene (αP) | 0.87 | 0.01 | 0.55 | 0.01 | 0.63 | 0.01 |
| β-pinene (βP) | 0.87 | 0.01 | 0.56 | 0.01 | 0.90 | 0.01 |
| β-phellandrene (βL) | 0.87 | 0.01 | 0.48 | 0.01 | 0.78 | 0.01 |
| Camphene (CM) | 0.84 | 0.01 | 0.45 | 0.01 | 0.47 | 0.01 |
| Limonene (LM) | 0.51 | 0.01 | 0.88 | 0.01 | 0.58 | 0.01 |
| Bornyl acetate (BA) | 0.50 | 0.01 | 0.19 | 0.23 | 0.02 | 0.97 |
| Terpinolene (TR) | 0.49 | 0.01 | 0.40 | 0.01 | 0.19 | 0.16 |
| Myrcene (MY) | 0.45 | 0.01 | 0.54 | 0.01 | 0.62 | 0.01 |
| *p*-cymene (CY) | 0.42 | 0.01 | 0.57 | 0.01 | 0.14 | 0.41 |
| 3-carene (3C) | 0.40 | 0.01 | 0.60 | 0.01 | 0.29 | 0.07 |
| γ-terpinene (γT) | 0.34 | 0.01 | 0.52 | 0.01 | 0.01 | 1.00 |
| α-terpinene (αT) | 0.32 | 0.01 | 0.47 | 0.01 | 0.14 | 0.37 |
| Camphor (CP) | 0.21 | 0.12 | 0.13 | 0.58 | 0.20 | 0.22 |
| 4-Allylanisole (4A) | 0.21 | 0.17 | 0.23 | 0.11 | 0.14 | 0.42 |
