# Supplementary figures and images for: Drought stress leads to systemic induced susceptibility to a nectrotrophic fungus associated with mountain pine beetle in Pinus banksiana seedlings
Source: PLoS One. 2017 Dec 7;12(12):e0189203. doi: 10.1371/journal.pone.0189203 (PMC5720781; doi:10.1371/journal.pone.0189203)

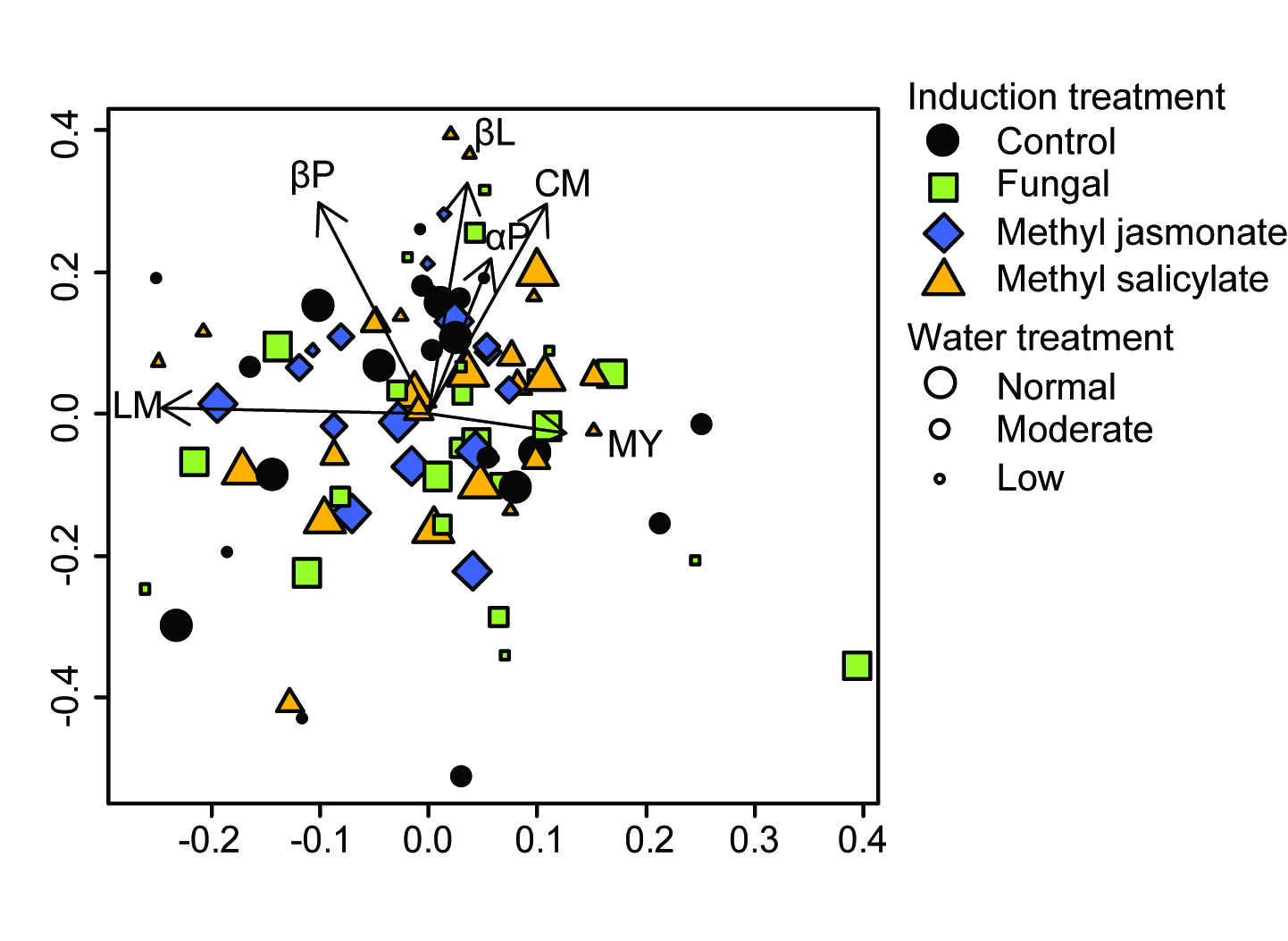

Supplement: S1 Fig — Non-metric multidimensional scaling with Bray-Curtis distance ordination was used to analyze relationships. Significant monoterpene compounds are represented by overlaid vectors with direction indicating association with corresponding induction and watering treatments (α = 0.05). Longer vectors show stronger correlations with the ordination configuration (i.e., axes 1 and 2). The minimum stress was 0.14. See S2 Table for abbreviations for monoterpenes, correlations, and P-values. (TIF) [file pone.0189203.s003.tif]
